# Accuracy of echocardiography and ultrasound protocol to identify shock etiology in emergency department

**DOI:** 10.1186/s12873-022-00678-6

**Published:** 2022-06-30

**Authors:** Asmaa Ramadan, Tamer Abdallah, Hassan Abdelsalam, Ahmed Mokhtar, Assem Abdel Razek

**Affiliations:** 1 Department of Emergency Medicine, Alexandria University, Alexandria, Egypt; 2 Department of Critical Care Medicine, Alexandria University, Alexandria, Egypt; 3 Department of Radiology, Alexandria University, Alexandria, Egypt; 4 Department of Cardiology and angiology, Alexandria University, Alexandria, Egypt; 5 Department of Anesthesia and Surgical Intensive Care Unit, Alexandria University, Alexandria, Egypt

**Keywords:** Accuracy, Echocardiography, Ultrasound, Shock

## Abstract

**Background:**

Early diagnosis and appropriate management of shock aimed at prevention of prolonged hypoperfusion has shown to decrease morbidity and mortality in patients with undifferentiated shock. However, there is often a challenge in emergency department (ED) – where diagnosis is mainly based on clinical signs and standard monitoring parameters. Early use of point of care ultrasound could reduce the diagnostic time and improve diagnostic accuracy.

**Purpose:**

The aim of this study is to investigate the accuracy of echocardiography - ultrasound protocol to identify the cause of shock in ED.

**Method:**

The study was conducted on 150 shocked patients admitted to emergency department of Alexandria Main University Hospital from December 2018 to December 2020. The study was conducted to reach initial impression about shock etiology which was then compared to final diagnosis to determine accuracy, agreement, sensitivity, specificity, positive predictive value (PPV) and negative predictive value (NPV).

**Results:**

One hundred forty patients were included in the study (10 patients were excluded). The protocol was 100% accurate for diagnosing cases with obstructive and mixed obstructive distributive shock. It showed excellent rule- out characteristics for cardiogenic shock (sensitivity and NPV = 100%). There was almost perfect agreement between provisional and final shock type for mixed distributive cardiogenic shock (kappa 0.915). Echo- US protocol had lowest agreement and PPV for patients with hypovolemic shock Kappa 0.48 and 35% respectively.

**Conclusion:**

The Echo- US protocol showed a high accuracy in identifying shock etiology in ED and is likely a promising diagnostic tool in emergency care.

## Background

Shock is a life threatening condition in which there is insufficient oxygen delivery to tissues to meet metabolic demand [1]. Early diagnosis and appropriate management aimed at prevention of prolonged hypoperfusion has shown to decrease morbidity and mortality [2]. However, there is often a challenge in emergency department (ED) depending mainly on clinical signs and standard monitoring parameters. The first ultrasound protocol for undifferentiated hypotension was published on 2001 and subsequently more than 15 protocols were proposed [3].

There is significant overlap of clinical findings in different types of shock and on the other hand laboratory investigations are time consuming. This is even more challenging in critically ill patients [4].

To overcome this, point of care protocols integrate focused cardiac examination, inferior vena cava (IVC), aorta and lung ultrasound to assist diagnosis. The goal is to find a systematic way to classify the non-specific clinical syndrome into more specific types of shock [5].

Echocardiography is a promising method for initial management of shocked patients; it also allows obtaining a more complete hemodynamic picture. Moreover, there is increasing evidence showing the association between echocardiography in shock and improved outcome reported in observational studies [6]. However, there is limited evidence for this approach in emergency care.

There are a lot of studies evaluating accuracy of ultrasound for shock diagnosis but most of them had small sample size as Bagheri et al. [7], Ghane et al. [8] and Vaidya et al. [9] which included 25, 52 and 100 patients respectively. Almost all studies of ultrasound in shock assess the Rapid Ultrasound in Shock (RUSH) protocol with a common finding: lowest agreement, sensitivity, specificity and accuracy were for distributive shocks. Moreover, RUSH exam could not differentiate between hypovolemic shock and early septic shock. We utilized Left Ventricular Out flow Tract Velocity Time Integral (LVOT VTI) as surrogate for stroke volume and cardiac output to differentiate distributive from other types of shock as it is low in all types of shock except distributive. We combined ultrasound findings of pneumonia to increase the diagnostic accuracy. All studies regarding the use of ultrasound in shock relied on reduced left ventricular function to diagnose cardiogenic shock, with no attention to other etiologies such as valvular pathologies or mechanical complications of myocardial infarction.

In this study, we aimed to investigate the accuracy of echocardiography - ultrasound protocol to identify the cause of shock in ED.

## Patients and methods

### Patients

This cross sectional study was conducted on a convenience sample of 150 patients from December 2018 to December 2020 in emergency department of Alexandria Main University Hospital - a major university hospital and a tertiary care center in the north of Egypt. All shocked patients admitted to the hospital during working shifts of the researcher (36 hrs per week) who met the criteria for the study were enrolled. Patients with high body mass index and patients with poor echo view such as patients with hyper inflated chest were also included in the study with no selection bias.

The inclusion criteria were: adult patients with shock (defined as SBP < 90 mmHg or shock index ≥1 with clinical signs of tissue hypo perfusion). Patients with an obvious cause of shock as gastrointestinal bleeding, patients with tacchy- or bradyarrhythmia, burn, trauma, post arrest and patients who received intravenous fluids before hospital admission were excluded from the study.

Ethical approval was granted from Alexandria University Ethical Committee (Reference number 0201184) Informed consent was taken from all patients or next of kin.

### Machines used

Echocardiography was carried out using Vivid e machine (General Electric, Boston, USA) with 2.8–4 MHz phased array probe and one of three ultrasound devices: Mindray DC-30, DP-5 and DP-20 (Mindray, Shenzhen, China) with a curvilinear probe (2–5 MHz) and a high frequency linear probe (5–10 MHz).

All ultrasound examinations were carried out by the first author, AR – emergency specialist certified from the Egyptian Medical Society of Echocardiography with 5 years’ experience in emergency ultrasound. The operator had attended a minimum of 110 hours dedicated clinical ultrasonography, 20 hours of didactic ultrasonography education that covered all emergency ultrasound applications also, had performed more than 500 reviewed echocardiography and ultrasound examinations before conducting the study.

US exam was carried out without interruption or delay in patient care also, the duration of the whole scan was recorded. Time to reach final diagnosis was also documented.

**Fig. 1:**
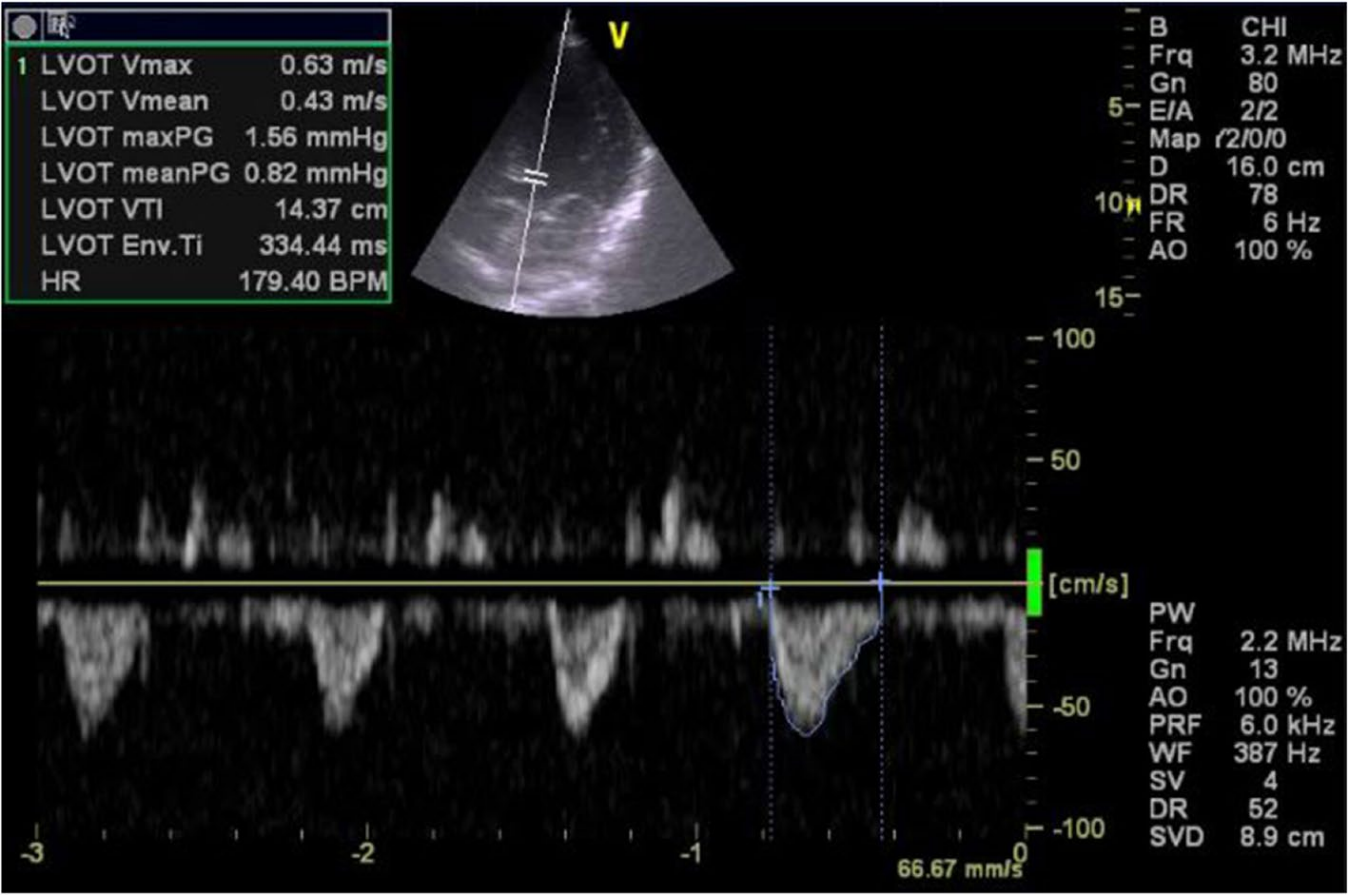
Apical 5 chamber view demonstrating lowAortic VTI =14.37 cm in patient with cardiogenic shock

**Fig. 2:**
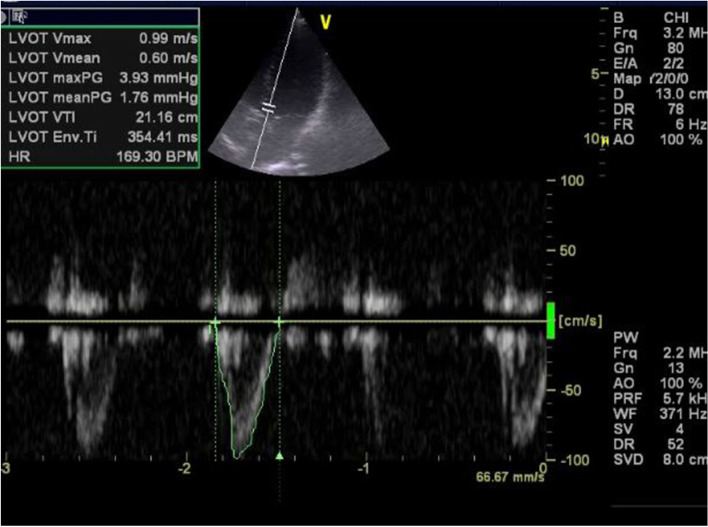
Apical 5 chamber view demonstrating Aortic VTI = 21.16 cm in patient with septic shock

The following echocardiography/ultrasound parameters were recorded:

- Left ventricular outflow tract velocity time integral (LVOT VTI) as in Figs. 1 and 2. If LVOT VTI could not be obtained mitral VTI was used instead [6].
- Left ventricular ejection fraction (EF) was calculated by M-mode, in case of difficulty in getting parasternal view or in case of regional wall motion abnormality eyeballing method was used instead.
- Mechanical complications of myocardial infarction.
- Signs of cardiac tamponade
- Signs of pulmonary embolism
- Valves for infective endocarditis, stenosis or regurgitation
- Aorta to detect aortic dissection or rupture aneurysm.
- IVC size, collapsibility and distensibility index were calculated [10, 11] In case of unvisualized IVC, internal jugular vein (IJV) was used instead.
- Focused Assessment with Sonography for trauma (FAST) exam.
- Compression test using ultrasound to detect lower extremity deep vein thrombosis (DVT).
- Lung ultrasound to detect of pneumothorax, pleural effusion, pneumonia or pulmonary edema.

All these data were utilized to reach a provisional diagnosis of shock type based on data presented in Table 1.

**Table 1:** Echo – US protocol findings in each type of shock for typical cases

	Distributive	Hypovolemic	Cardiogenic	Obstructive	Mixed distributive cardiogenic	Mixed distributive obstructive
Echo						
VTI Aorta	≥ 18 cm	< 18	< 18	< 18	Variable	Variable
LV systolic function	Hyper dynamic/ Normal	Hyper dynamic	Reduced	Hyper dynamic	Reduced	Hyper dynamic
IVC	Small	Small	Large	Large	Variable	Variable
	CI ≥50%	CI ≥50%	CI < 50%DI < 1	CI < 50%		
	DI ≥18%	DI ≥18%	8%	DI < 18%		
Pulmonary embolism	NO	NO	NO	YES	NO	YES
Valves	Vegetation	Normal	Stenosis / Regurgitation	TR in PE	Stenosis /Regurgitation	Normal/ Regurgitation
Tamponade	NO	NO	NO	YES	NO	YES
Aorta	Normal	Rupture AAA	Normal	Normal	Normal	Normal
FAST						
FAST	Positive / Negative	Positive / Negative	Positive / Negative	Negative	Positive / Negative	Positive / Negative
CUST						
DVT	Negative	Negative	Negative	Positive	Negative	Negative
Lung U/S						
Pneumothorax	NO	NO	NO	YES	NO	YES
Pneumonia	YES	NO	NO	NO	YES	YES
Pulmonary edema	NO	NO	YES	NO	YES /NO	NO
Pleural effusion	Present/ absent	Absent	Present/ Absent	Absent	Present/ Absent	Present/ Absent
IJV						
IJV	Small / at neck root	Small / at neck root	Large/ At angle of mandible	Large/ At angle of mandible	Variable	Variable

*AAA* abdominal aortic aneurysm, *CI* collapsibility index, *CUST* compression test using ultrasound, *DI* distensibility index, *DVT* deep vein thrombosis, *FAST* Focused assessment with sonography for trauma, *IJV* internal jugular vein, *IVC* inferior vena cava, *LV* left ventricle, *PE* pulmonary embolism, *TR* tricuspid regurgitation, *U/S* ultrasound, *VTI* velocity time integral

All patients were followed during their hospitalization period in order to document their final diagnosis. Final diagnosis was established by external committee (not involved in the study) whom the patient was transferred to (Intensive Care unit, Surgical Unit, Cardiology Department or Internal Medicine Unit). Since there was no suitable comparative diagnostic tool existing across etiologies, reference standard for each shock etiology was used which includes Echo by cardiologist for cardiac tamponade, acute massive pulmonary embolism, and cardiogenic shock. There is no gold standard test for diagnosis of septic shock as in surviving sepsis campaign guidelines 2021 several laboratory investigations and imaging modalities as (Complete blood count, C- reactive protein, cultures, biological fluid analysis, ultrasound abdomen, CT chest, CT abdomen and pelvis) were utilized. Echocardiographic findings were revised by the fourth author AM- cardiology consultant also; ultrasound results were compared to CT scans or ultrasound results which were done by certified radiologists.

Data were collected and entered into SPSS program (IBM, Chicago, USA) for statistical analysis (version 21) [12]. Kolmogorov-Smirnov test of normality was used and non-parametric tests were used accordingly [13]. Data were presented as minimum, maximum, median and inter-quartile range.

Cohen’s kappa and weighted Cohen kappa coefficients (κ) were used to estimate inter-rater agreement for qualitative parameters [14, 15]. For the assessment of kappa coefficient, Landis and Koch [16] magnitude guidelines for agreement were used. Two by two tables were constructed and sensitivity, specificity, positive predictive value (PPV), negative predictive value (NPV) and accuracy were calculated [17].

The minimum sample size required was 135 patients to detect a sensitivity of 90% with precision of 10% and alpha of 0.05. Assuming around 10% rate of missing data a sample size of 150 patients was required.

## Results

We enrolled 150 patients 78 males were (52%) and 72 were female (48%). Ten patients were excluded from the study (6 patients died before reaching final diagnosis and 4 patients discharged against medical advice before reaching final diagnosis). The median age was 59.5 years (Interquartile range, IQR, 46–73). The median systolic blood pressure (SBP) was 80 mmHg (IQR 70–90). The median diastolic blood pressure (DBP) was 50 mmHg (IQR 40–50). Forty two cases had unrecorded SBP by noninvasive method while, 44 cases had unrecorded DBP as in Table 2.

**Table 2:** Demographic and clinical characteristics of all enrolled patients

	Number	Range	Median	Interquartile range
Sex n (%)	150			
Male		78 (52%)	–	–
Female		72 (48%)		
Age (years)	150	20–96	59.5	46–73
Systolic blood pressure (mmHg)	108	50–110	180	70–90
Diastolic blood pressure (mmHg)	106	30–70	50	40–50
Mean blood pressure (mmHg)	106	36.7–83.3	60	50–63.3
Heart rate (bpm)	150	40–150	100	90–120
Initial lactate (mmol/L)	150	0.20–15	3.7	2.2–7.1

It was noted that not all ultrasound findings were obtainable from all patients as in Table 3. LVOT VTI was measured in 132 (88%) cases, while mitral VTI was measured in 11 cases (7.33%) as the pulsed wave Doppler could not be placed parallel to LVOT blood flow. Aortic or mitral VTI could not be measured in seven cases due to presence of valve pathology, difficult views or patients presented in near arrest situation where lifesaving intervention as pericardiocentesis was performed immediately. CUST could not be done in one patient due to presence of groin abscess.

**Table 3:** Echocardiography and ultrasound finding of the studied cases (150 patients)

Parameters	Number of cases tested	Findings (number & Percentage)
LVOT VTI	132 (88%)	< 18 cm: 64 (42.67%)
		≥ 18 cm: 68 (45.33%)
VTI mitral	11 (7.33%)	< 10 cm: 1 (0.67%)
		≥ 10 cm: 10 (6.67%)
LV systolic function	150 (100%)	Normal: 46 (30.67%)
		Hyperdynamic: 69 (46%)
		Reduced: 35 (23.33%)
IVC maximal diameter, IVCCI or IVCDI	134 (89.33%)	< 1.5 cm: 65 (43.33%)
		1.5–2.1 cm: 26 (17.33%)
		> 2.1 cm: 43 (28.67%)
		IVCCI< 50%: 59 (39.33%)
		IVCCI ≥50%:52 (34.67%)
		IVCDI< 18%: 10 (6.67%)
		IVCDI≥18%: 13 (8.67%)
Infective endocarditis	150 (100%)	Present: 3 (2%)
		Absent: 147 (98%)
Tamponade	150 (100%)	Present: 3 (2%)
		Absent: 147 (98%)
Pulmonary embolism	150 (100%)	Present: 3 (2%)
		Absent: 147 (98%)
Aorta	Abdominal: 129 (86%)	AAA: 2 (1.33%)
	Thoracic: 128 (85.33%)	Thoracic aortic dissection: 1 (0.66%)
	Arch: 91 (60.67%)	
FAST	150 (100%)	Positive: 39 (26%)
		Negative: 111 (74%)
CUST	149 (99.33%)	Positive: 15 (10%)
		Negative: 134 (89.33%)
Pneumothorax	150 (100%)	Present: 3 (2%)
		Absent: 147 (98%)
Pneumonia	150 (100%)	Present: 50 (33.33%)
		Absent: 100 (66.33%)
Pulmonary edema	150 (100%)	Present: 21 (14%)
		Absent: 129 (86%)
Pleural effusion	150 (100%)	Present: 31 (20.67%)
		Absent: 119 (79.33%)
IJV	16 (10.67%)	Small at neck root: 14 (9.33%)
		Large at angle of mandible: 2 (1.33%)

*IVCCI* inferior vena cava collapsibility index, *IVCDI* inferior vena cava distensibility index

There was almost perfect agreement between provisional and final shock type for patients with cardiogenic shock (kappa 0.842) with 100% sensitivity and negative predictive value as shown in Tables 4 and 5. Echo assessment for patients with cardiogenic shock revealed all cases had reduced ejection except one case who had EF = 78.7%. This case had acute severe mitral regurgitation due to rupture chordae as complication of acute myocardial infarction. One patient had severe mitral stenosis and another patient had both aortic stenosis and regurgitation as an etiology for cardiogenic shock.

**Table 4:** Provisional versus final shock type for all types of shock

**Provisional shock type**	**Final shock type**							
		Distributive	Cardiogenic	Hypovolemic	Obstructive	Mixed Distributive cardiogenic	Mixed distributive obstructive	Total
	Distributive	83	0	0	0	0	0	83 (59.28%)
	Cardiogenic	2	12	0	0	2	0	16 (11.43%)
	Hypovolemic	13	0	7	0	0	0	20 (14.29%)
	Obstructive	0	0	0	4	0	0	4 (2.86%)
	Mixed Distributive cardiogenic	0	0	0	0	12	0	12 (8.57%)
	Mixed distributive obstructive	0	0	0	0	0	5	5 (3.57%)
	Total	98 (70%)	12 (8.57%)	7 (5%)	4 (2.86%)	14 (10%)	5 (3.57%)	140 (100%)

**Table 5:** Diagnostic test parameters for Echo –US protocol for each shock type

	Distributive	Cardiogenic	Hypovolemic	Obstructive	Mixed distributive cardiogenic	Mixed distributive obstructive
Sensitivity	84.69%	100%	100%	100%	85.71%	100%
Specificity	100%	96.88%	90.23%	100%	100%	100%
PPV	100%	75%	35%	100%	100%	100%
NPV	73.68%	100%	100%	100%	98.44%	100%
Accuracy	89.29%	97.14%	90.71%	100%	98.57%	100%
Agreement	0.769	0.842	0.48	1	0.915	1

*PPV* positive predictive value, *NPV* negative predictive value

There is almost perfect agreement between provisional and final shock type for patients with obstructive shock (kappa = 1). Three patients had pulmonary embolism and one patient had cardiac tamponade. Sensitivity, specificity, PPV, NPV and accuracy of the protocol for diagnosis of patients with obstructive shock was 100%.

There was moderate agreement between provisional and final shock type for hypovolemic shock (kappa 0.480) with 100% sensitivity and negative predictive value. There was substantial agreement between provisional and final shock type for distributive shock (kappa 0.769) with 84.69% sensitivity and 100% specificity.

There was almost perfect agreement between provisional and final shock type for mixed distributive obstructive shock (kappa = 1) with 100% Sensitivity, specificity, accuracy, PPV and NPV. Three cases had tension pneumothorax and two cases had cardiac tamponade. All cases had pneumonia and also two cases had empyema as underlying septic etiology.

There was almost perfect agreement between provisional and final shock type for mixed distributive cardiogenic shock (kappa 0.915). Sensitivity of echo –US protocol was 85.7% with 95% CI (57.19 to 98.22%) with specificity and PPV of 100%.

The duration of our echo- ultrasound protocol ranged from 7.17 minutes to 27.21 minutes with a median duration of 16.74 minutes. Echo-US protocol deduced the diagnosis in 135 patients before standard method with median time of 2.50 hrs (IQR 1.75–3.75 hrs). On the other hand, final diagnosis was deduced in five cases before the protocol was applied.

## Discussion

In this study we demonstrated high diagnostic accuracy for the Echo-US protocol performed by an emergency physician to identify the type of shock in ED. When compared with the final inpatient diagnosis, our protocol had an accuracy ranging between 89.29 and 100%. This suggests a place for more extended Echo-US (beyond traditional basic scanning) as part of clinical assessment in ED.

The protocol showed excellent rule- out characteristics for cardiogenic shock (sensitivity and NPV = 100%). It also had excellent agreement with Kappa index of = 0.842. In a similar study by Ghane et al. [18] showed sensitivity of 91.7%, NPV (97%) and kappa index = 0.89. Important causes of cardiogenic shock detected by our protocol included acute mitral regurgitation due to rupture chordae, mitral stenosis, aortic stenosis and regurgitation thus; valve assessment in any shocked patient should be emphasized.

This protocol showed highest accuracy in obstructive shock. These results are comparable to the work done by Ghane et al. [8], demonstrating sensitivity of 100%; specificity = 97%, PPV 87.5%, NPV 100% and Kappa index = 0.92. This also agrees with a study done by Vaidya et al. [9] which found maximum sensitivity, specificity, PPV, NPV for obstructive shock with a kappa index of =1. This may be attributed to the fact that echo signs of massive pulmonary embolism were evident and ultrasound finding of pneumothorax were easily identified.

A common ED diagnostic dilemma is to differentiate between hypovolemic and distributive shock and consequently, when to stop fluids and start vasopressor therapy. Differentiation between these types of shock by different ultrasound protocols was not easy as both types had normal or hyperdynamic LV function, small collapsible IVC and positive FAST. With the addition of LVOT VTI and ultrasound finding of pneumonia sensitivity for detection of distributive shock increased to 84.69%. This was higher than the results shown by Keikha et al. [19] and Ghane et al. who reported sensitivity of 73 and 75% respectively.

For patients with distributive shock, 64.77% of them had LVOT VTI ≥ 18 cm while 35.23% had LVOT VTI <  18 cm which was statistically significant (p _(MC)_ = 0.000) because distributive shock had normal or increased cardiac output unlike other types of shock which had reduced cardiac output. This is may be attributed to patients present at different pathophysiological changes with variable cardiac output and subsequent VTI. Moreover, some cases were severely volume contracted which resulted in low VTI, whilst others had preexisting ischemic heart disease with subsequently low cardiac output.

Hypovolemic shock showed Kappa index of 0.48, which was lower than other studies done by Bagheri et al. [7] and Ghane et al. [18] with kappa index 0.75 and 0.92 respectively. This lower agreement with the present study is due to some cases showing initial impression of hypovolemic shock according to the protocol which then proved to be septic shock in final diagnosis.

Our protocol was feasible to all patients with a median time of 16.7 minutes. This is longer than the duration of scan in a study carried out by Rahulkumar et al.^(20)^ with mean time of 12 min. Obese patients and patients with poor echo and ultrasound views were not excluded from the study which resulted in longer exam time (> 20 minutes) in 15.7% of cases. Scanning such patients is often a challenge and time consuming but this improved the diagnostic certainty.

The Echo-US protocol had identified 55 patients (39.3%) where the protocol was more sensitive in early determining of the etiology and thus had their management altered to target the newly identified conditions.

Patients with cardiogenic shock two cases had severe valve lesion and one case had mechanical complication of acute myocardial infarction which would have been treated improperly without the use of Echo-US protocol.

One patient came in near arrest circumstances where pericardiocentesis was done immediately as a lifesaving intervention and complete assessment demonstrated presence of mixed shock etiology.

Echo- US protocol had solved the dilemma where the clinical picture overlap, as three middle age male patients presented with dyspnea, shock and history of intravenous drug use. This study revealed septic shock etiology due to chest infection and infective endocarditis in the first case; mixed septic- obstructive shock due to pneumothorax and empyema in the second case; and the last case had mixed septic cardiogenic shock. Three cases presented with shock and abdominal pain where one of them was identified with intraabdominal hemorrhage, while the other cases had ruptured abdominal aortic aneurysm.

Two cases of acute pulmonary embolism would have been misdiagnosed without this protocol as one case had initial impression of septic shock and the other case as cardiogenic shock.

For patients with mixed septic cardiogenic shock (10% of cases) early use of echo in these cases resulted in more caution administration of fluid.

Underlying source of sepsis such as infective endocarditis, chest infection and intraabdominal source were identified in 44% of cases with septic shock and subsequent initiation of specific therapy.

In this study, we highlighted the role of an extended Echo-US protocol as a potentially accurate method for rapid diagnosis of shock etiology in ED. While basic ultrasound is an integral part of emergency medicine practice in many settings, there is likely room for more advanced Echo- US techniques in emergency care. The paradigm should be shifted to more advanced echocardiography and ultrasound scan which are feasible as they are goal directed rather than comprehensive. Emergency physician should be competent in image acquisition and interpretation of all parameters used in this protocol and able to integrate different findings together. This requires didactic lessons, hands on sessions and review of examinations until the emergency physician can safely integrate these skills.

Future research should focus on the emergency physician learning curve of these techniques; inter-rater reliability; and impact on the ED decision making and patient related outcomes.

## Limitation

The present study looked at a convenience sample of ED patients during working shift of the operator. It was carried out by a single operator; therefore we cannot comment on inter-rater reliability of the protocol. Small number of patients was included in some of the shock subgroups which may have impacted the precision of results.

## Conclusion

An Echo - US protocol showed a high accuracy in identifying shock etiology in ED and is likely a promising diagnostic tool in emergency care. Learning curve and inter- rater reliability for this protocol will be important areas to explore prior to translating results into practice.

## Data Availability

The datasets generated and/or analyzed during the current study are not publicly available due privacy and data safety but are available from the corresponding author on reasonable request.
